# *MEDIotWALL*: Securing Smart Healthcare Environments Through IoT Firewalls

**DOI:** 10.3390/s25196235

**Published:** 2025-10-08

**Authors:** Irene Gosálvez-White, Nerea Rodríguez-Martín, Nicolás Barajas-García, Carmen Mena-Gallardo, Pedro García-Teodoro

**Affiliations:** 1 Network Engineering & Security Group, School of Computer Science and Telecommunications Engineering, University of Granada, 18071 Granada, Spain; nerearodrimar@correo.ugr.es (N.R.-M.); pgteodor@ugr.es (P.G.-T.); 2 Health Andalusian Service, 18071 Granada, Spain; nicolas.barajas.sspa@juntadeandalucia.es (N.B.-G.); carmen.mena.sspa@juntadeandalucia.es (C.M.-G.)

**Keywords:** healthcare, IoMT, firewall, IDS

## Abstract

IoT technology is transforming the healthcare industry through what is known as the Internet of Medical Things (IoMT), enhancing patient care while simultaneously reducing costs. Nevertheless, it introduces critical security challenges, particularly the risk of jeopardizing patient safety. Existing IoMT security solutions, often limited to proprietary platforms or generic IoT firewalls, frequently lack transparency, scalability, and awareness with clinical regulations. To address these gaps, we present *MEDIotWALL*, a customized two-tier security architecture tailored for healthcare environments. The system integrates distributed real-time traffic monitoring with AI-driven rule generation, delivering a non-intrusive, centralized, human-supervised, and regulation-aware security framework. Experimental results demonstrate the cost-efficiency and effectiveness of the approach, ensuring robust medical protection while preserving authentication, confidentiality, and integrity of the environment.

## 1. Introduction

**Figure 1:**
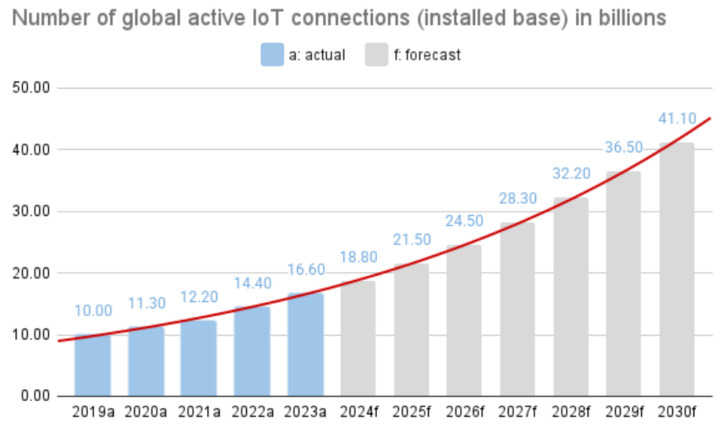
Global IoT market forecast (billion of connected IoT devices) [2].

As shown in several studies, such as [1,2,3], the number of IoT (Internet of Things) devices in use has increased from 10 billion in 2019 to around 18.8 billion by the end of 2024. With the advancement of 5G and other technologies, this number is projected to more than double, reaching 41.1 billion devices by 2030 (see Figure 1). Consequently, the total IoT annual revenue worldwide is expected to grow from 369 billion U.S. dollars in 2024 to 908 billion U.S. dollars by the end of 2034.

The rapid expansion of the IoT is fueling a digital transformation that surpasses the combined influence of the personal computer and mobile revolutions—impacting all sectors, including healthcare. This evolution, often referred to as the Internet of Medical Things (IoMT) or Healthcare IoT (H-IoT), is poised to significantly reshape the medical device industry. Projections estimate the market will reach USD795 billion by 2030, driven by a compound annual growth rate (CAGR) of 5.2% between 2015 and 2030. Market research firm IDC (International Data Corporation) anticipates that by 2025, approximately 42 billion connected devices will generate 79.4 zettabytes of data [4].

While the integration of IoMT brings promising benefits—such as improved patient care, more accurate clinical data, increased operational efficiency, and reduced costs—it also introduces significant security challenges. These risks have the potential to endanger patient safety and compromise sensitive medical information [5].

**Figure 2:**
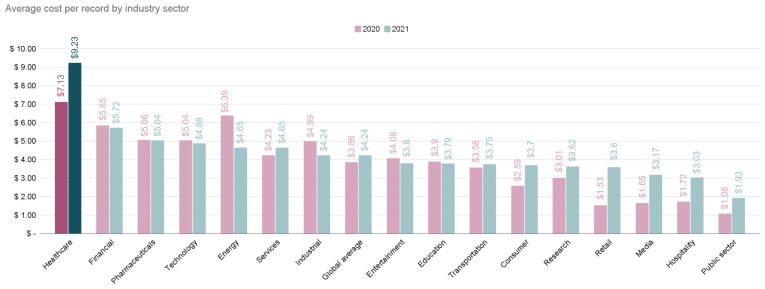
Average cost per compromised record by industry sector [6].

According to a study conducted by the Ponemon Institute [6], the average total cost of a data breach in the healthcare sector was around USD9.23 million in 2022 (see Figure 2).

According to different reports like [7,8,9], some relevant figures regarding healthcare security are as follows:

- A total of 64% of healthcare delivery organizations (HDOs) have more IoT devices connected to their networks than computers.
- In a typical hospital, over 50% of connected devices carry significant risks.
- Nearly 75% of IV pumps have vulnerabilities that could endanger patient safety if exploited.
- More than half of the devices in oncology, pharmacology, and laboratory departments are running outdated versions of Windows that are no longer supported.
- A total of 63% of HDOs have encountered at least one security incident involving unmanaged or IoT devices.
- While new vulnerabilities attract attention, insecure passwords remain the most common device risk.
- Around 80% of security managers in HDOs believe their current spending on IoT device security is insufficient.
- Over 80% express serious concern about the risks associated with IoT devices.

Given these growing concerns, healthcare providers must prioritize not only the prevention of data breaches and information leaks but also the safeguarding of patient safety [10]. In response to this complex and pressing challenge, regulatory and compliance frameworks such as HIPAA (Health Insurance Portability and Accountability Act) and HITRUST (Health Information Trust Alliance) have been introduced to enhance security within healthcare environments [11].

The HIPAA is a U.S. federal law that establishes national standards for the security, privacy, and proper management of protected health information (PHI). It applies to covered entities—including healthcare providers, insurance companies, and healthcare clearinghouses—as well as their business associates, such as third-party service providers and software vendors operating in these sectors [12,13].

To further support these efforts, the HITRUST, a non-profit organization founded in 2007, developed the HITRUST Common Security Framework (CSF). Created in collaboration with stakeholders from the healthcare, technology, and information security industries, the HITRUST CSF offers a comprehensive, harmonized approach to managing compliance. It integrates and streamlines more than 40 existing security standards, frameworks, and regulations into a single, unified framework [14].

In this context, the present work introduces *MEDIotWALL*, a dynamic, two-level IoT-based security solution tailored for medical environments. The architecture consists of IoMT devices, dedicated firewalls that monitor these devices, and a central management entity responsible for supervising and coordinating the entire system. The core operation is supported on the firewalls, which suggest potential security rules for the devices while, simultaneously, enforce additional rules determined by the central management entity to protect the overall environment. *MEDIotWALL* constitutes a novel, low-cost and efficient proposal within the existing literature, with the following main features and contributions:

- It is supported on a two-tier approach, with two main security-related components: (a) a set of low-cost Raspberry Pi-based distributed firewalls, with each IoMT device (e.g., MRI machines, infusion pumps, incubators) in the environment assigned to a dedicated firewall; and (b) a web-based central unit, responsible for overseeing and managing the entire environment.
- According to this two-level architecture, the approach considers two separate monitoring procedures: (a) one central, where integral platforms like Claroty can be considered, and (b) a distributed one, associated with each of the firewalls around the environment.
- Automatic AI-based generation of traffic filtering rules to respond to the alerts provided from the firewalls associated with each of the IoMT devices.

According to the above, the organization of the paper is as follows: After the present Section 1, mainly intended to introduce the topic and its relevance, Section 2 discusses main proposals described in the specialized literature to provide security in IoMT environments. Section 3 presents the specific firewall solution developed by the authors: *MEDIotWALL*. Afterward, Section 4 presents the scenario deployed to evaluate the solution, the main experimental results being subsequently discussed. Finally, Section 5 concludes the main contributions and benefits of our security solution.

## 2. Background

According to the U.S. Department of Health and Human Services, the most significant cybersecurity attacks in healthcare today include the following [4]: email phishing, ransomware, loss or theft of equipment or data, insider threats (both accidental and intentional), and attacks targeting connected medical devices that could impact patient safety. These attacks often involve a combination of multiple threat types. Vulnerabilities such as TLStorm, Log4j, and URGENT/11 are among those that can directly compromise healthcare environments [6].

Several notable recent healthcare attacks are explored in [15,16]. Additionally, as highlighted in [10], such attacks can compromise patient safety in multiple ways. For instance, a study published in the New England Journal of Medicine [17] examined the effect of ambulance delays caused by rerouting around marathons, showing a statistically significant rise in 30-day mortality rates due to the impact on patient care.

With the European Medical Device Regulations taking effect in May 2021, the cybersecurity of medical IoT devices has become a key focus for the entire healthcare industry. As a result, initiatives like [18,19] have been launched to introduce an architecture and a risk model for the healthcare sector. It is important to note that not only traditional medical devices such as infusion pumps, incubators, medical imaging systems, and cardiac pacemakers need protection, but also related devices like clinical monitors (e.g., heart rate, pulse oximetry, and electrocardiogram), smart patient rooms (e.g., smart beds and fall detectors), and patient experience devices (e.g., label printers, tablets, smart TVs, electronic whiteboards, and elevator systems) [6].

As is clear, implementing comprehensive and integrated healthcare security solutions is crucial. Some of the top IoT security solutions for healthcare include Armis, Claroty, Palo Alto, Asimility, and Ordr, among others [20,21]. These platforms generally offer features such as data encryption, intrusion detection, access control, secure data transmission and storage, device authentication, and real-time monitoring. These capabilities provide enhanced protection against data breaches and unauthorized access, while also ensuring compliance with regulations like HIPAA. As a result, these tools often form a robust cybersecurity solution that ensures proper protection of medical devices, efficient resource utilization, and effective maintenance of all connected devices.

**Figure 3:**
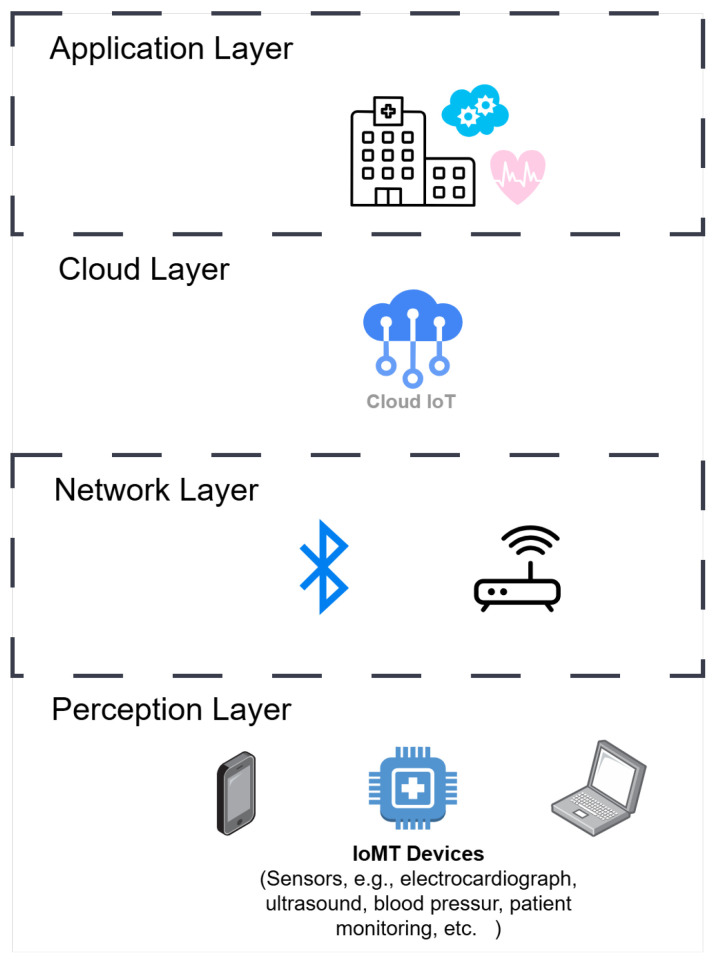
Generic IoT architecture [23].

Delving into the technical basis of IoT-related security solutions, the generally accepted architecture is composed of different layers [22,23] (see Figure 3):

- *Perception layer*: This refers to the physical level where sensors are deployed and involves the collection of information. The perception layer includes multiple communication protocols (i.e., Radio Frequency Identification, Bluetooth Low Energy, Wireless Sensor Networks, Zigbee, IPv6 over Low-Power Wireless Personal Area Networks) and is susceptible to multiple attacks affecting the infrastructure in various ways: jamming, eavesdropping, injection, cloning, tampering, etc.
- *Network layer*: This layer provides network transmission and information security, and delivers pervasive access environment to the perception layer. The network layer can be enabled with different wireless networks like Wi-Fi 6, 5G, Bluetooth, NB-IoT, and LTE, which are vulnerable to threats and attacks like spoofing, DDoS, routing, sybil, sinkhole, or Man-in-the-Middle.
- *Cloud layer*: To differentiate between transmission itself and intermediate storage, the network layer is sometimes divided so a fourth layer appears [23]: the *cloud* or *edge* layer. This allows secure backup and preservation of confidential health information, and sharing between authorized parties (doctors, insurance providers, medical staff, and pharmacies). In this case, attacks like flooding, web attacks and injection attacks may be deployed here.
- *Application layer*: This layer is visible to the end-user as it resides at the top level, and provides the user interface for each of the specific healthcare services. Attacks like phishing, DDoS (Distributed Denial of Service), buffer overflow, malware, XSS and code injection exist here.

In [23,24,25], the authors explore solutions to mitigate the aforementioned attacks. Specifically, the first of the three cited paper emphasizes Machine Learning approaches such as SVM, DT, RF, NN, and others. Similarly, studies like [26] examine prevention mechanisms for attacks across different IoT layers. More specifically, [27] presents defenses against perception-layer attacks; [28,29] explore mitigation strategies for cloud-layer attacks; and [30] discusses solutions for application-layer attacks.

In addition to the comprehensive IoT security solutions mentioned above, an essential element of IoT security is the IoT firewall [31,32,33]. This firewall plays a crucial role in protecting networked devices from online attacks by securing the data flow and communication between IoT devices and the broader network. Specifically, IoT firewalls monitor the data flow from connected devices to detect security threats, unusual patterns, or unauthorized access attempts. The key components of an IoT firewall include (a) packet inspection and filtering, (b) intrusion detection and mitigation, and (c) layer-based filtering for IoT protocols.

While traditional firewalls are designed to protect a single entry point, the diverse and continuous communication patterns of IoT devices can make monitoring and controlling traffic challenging, potentially leading to security weaknesses. This is where IoT device firewalls are essential, offering tailored protection for their intricate communication networks [34]. Most of the IoT firewalls available are proprietary solutions, such as Zenarmor, Cisco, Palo Alto Networks, Armis, Fortinet, and Check Point, which often come with a high cost.

In line with several proposals related to general sensor-based IoT environments [35,36,37,38], the following section introduces a low-cost IoMT firewall solution, *MEDIotWALL*, with the following key contributions:

- As a two-level solution, it comprises two main security-related components:
  − A set of Raspberry Pi-based firewalls, with each IoMT device (e.g., MRI machines, infusion pumps, incubators) in the environment assigned to a dedicated firewall.
  − A web-based central management unit, responsible for overseeing and managing the entire environment.
- The overall operation of *MEDIotWALL* can be summarized as follows:
  − Each IoMT device–firewall pair must be first activated through the central management unit. During this process:
    * Relevant security rules (e.g., nftables filtering rules) are identified for each IoMT device using a specialized centralized platform such as Claroty.
    * These rules are communicated to the corresponding firewall to ensure proper enforcement for the associated IoMT.
  − Additionally, each firewall monitors the traffic to/from its associated medical device over time so that
    * The traffic is locally analyzed by an IDPS (Intrusion Detection and Prevention System), and the potential alerts generated, if so, sent to the central management unit.
    * The alerts are studied and passed through an AI-based tool to automatically generate additional security rules at the central management unit, which should be manually confirmed by the administrator.
    * Upon confirmation, the rules are transmitted to the associated firewall for enforcement.

To the best of our knowledge, this dynamic security solution is novel in the existing literature and, as demonstrated below, offers a simple and cost-effective approach.

## 3. *MEDIotWALL*: An IoT Firewall Solution to Protect Medical Environments

While current IoMT security solutions provide proprietary platforms [20,21] or general IoT firewalls [34], they often lack transparency, scalability, and/or compliance to clinical regulations. *MEDIotWALL* is a customized low-cost, two-tier security architecture designed for healthcare environments, which integrates real-time traffic monitoring with AI-powered rule generation, delivering a distributed, non-intrusive, human-supervised, and regulation-aware security framework.

### 3.1. Functional Architecture

This section introduces the *MEDIotWALL*’s architecture, focusing on the integration of IoMT devices with secure management services. We also describe the operational flow that enables resilient deployment within hospital infrastructures, integrating automated security rule generation with centralized orchestration to ensure network policies are dynamically enforced and continuously aligned with clinical security requirements under strict human supervision.

**Figure 4:**
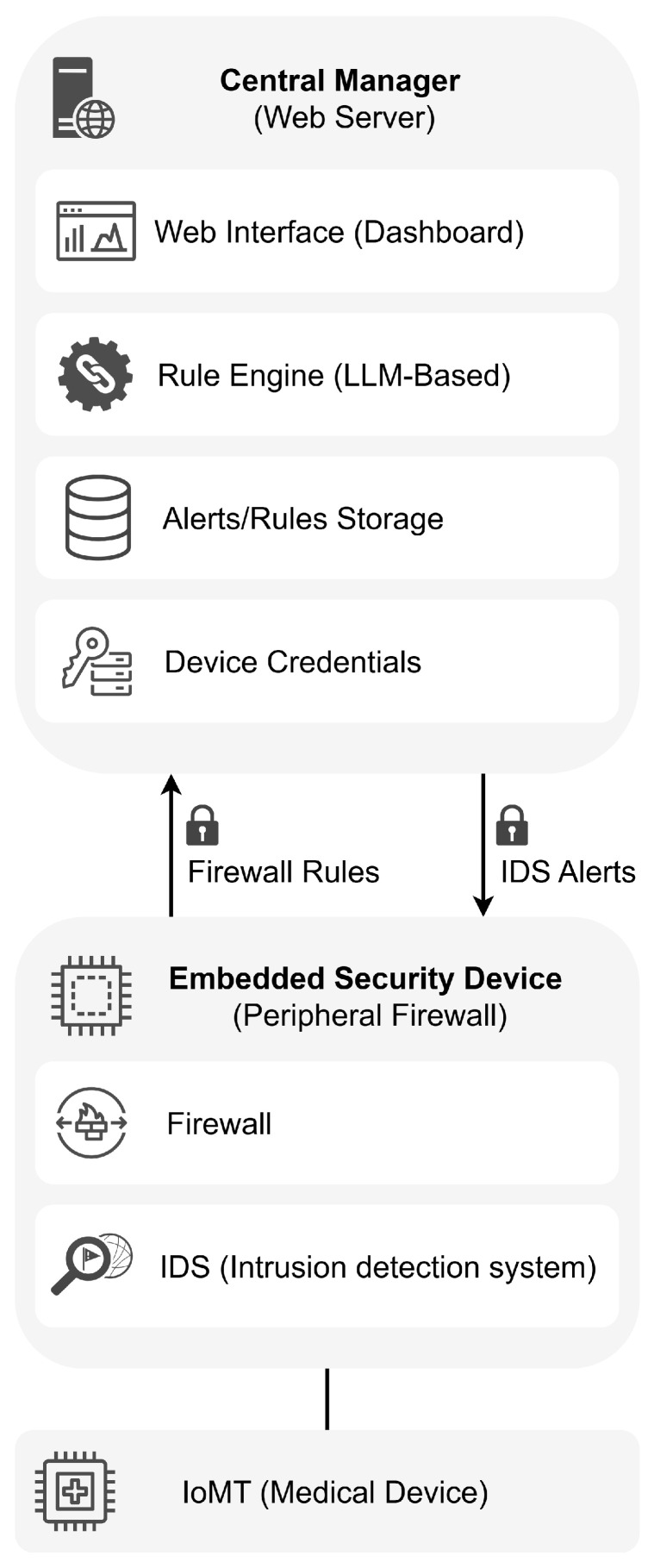
Main components of the *MEDIotWALL*’s functional architecture.

The proposed functional architecture is built on two core components that operate in coordination to safeguard IoMT medical devices while preserving their clinical operability (see Figure 4).

- *Central manager (Web server)*. This component serves as the core for system monitoring and control. Its primary functions include
  − Provision of a web interface for real-time monitoring of security events and alert management.
  − Reception of alerts transmitted by embedded security devices deployed across the hospital network.
  − Automatic analysis of alerts using Large Language Models (LLMs) to generate firewall rule suggestions based on detected threats and activities.
  − Filter and enable the remote application of firewall rules—subject to human approval—to prevent automated actions that could compromise the availability of medical devices.
  − Secure storage of the private keys of each device, ensuring the integrity and authenticity of communications with peripheral security devices.
- *Embedded security devices (ESDs, peripheral firewalls)*. Each critical medical device is protected by an embedded module that acts as a local firewall deployed transparently within the network. This module inspects and filters traffic without altering IP configurations or affecting device certification. Its capabilities include
  − Traffic inspection using an open-source intrusion detection system (IDS) to identify anomalous behavior or attack patterns.
  − Secure transmission of alerts to the central manager, protected with device-specific cryptographic keys to ensure integrity and authenticity.
  − Reception of updated rules with mutual authentication from the central manager, ensuring authorized configuration.
  − Traffic filtering based on centrally generated rules, which are applied locally through an open-source firewall.

Each embedded device is placed inline along the network path between the medical equipment and the hospital infrastructure, enabling traffic inspection and filtering without altering the IP configuration or impacting the device’s certification. This bump-in-the-wire topology (inline transparent deployment) ensures seamless, non-intrusive operation—an essential requirement in high-stakes healthcare environments.

**Figure 5:**
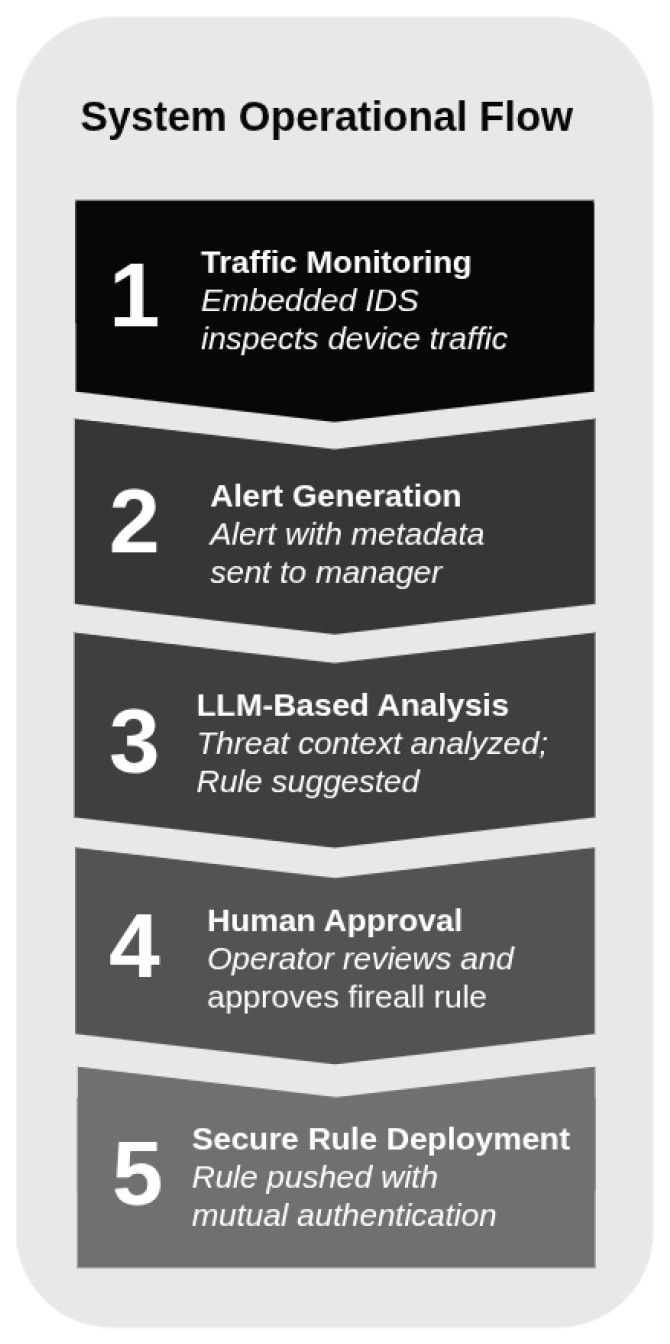
Security operational flow of *MEDIotWALL*.

The system operates through a structured sequence aimed at detecting, analyzing, and mitigating potential security threats while safeguarding the authenticity and integrity of clinical operations. This workflow integrates real-time monitoring with human oversight, instilling confidence in both medical staff and administrators (see Figure 5):

1. Local monitoring: Each embedded security module monitors network traffic from its associated medical device using an open-source IDS. This system detects anomalous behaviors or known attack patterns in real time without disrupting device operation.
2. Alert generation: Upon identifying suspicious activity, the local module generates an alert containing relevant metadata, including source/destination IP, ports or signature ID, and transmits it securely—protected with the device’s cryptographic key pair—to the central manager, ensuring authenticity and integrity.
3. Centralized alert analysis: The central manager processes the alert using a LLM, which analyzes the threat context and proposes a tailored firewall rule to mitigate the risk while preserving device functionality.
4. Human supervision: Proposed rules are announced to an authorized operator via the system’s web interface, requiring explicit human approval before implementation. This human-in-the-loop approach ensures accountability and protects patient safety.
5. Remote rule deployment: Approved rules are securely transmitted to the relevant embedded security module and applied using mutual authentication to verify both source and destination, ensuring a secure configuration update.

As a summary, the proposed approach marks a substantial advance over conventional firewall-based solutions by following design principles specifically adapted to the operational and regulatory constraints of healthcare environments, ensuring daily functionality while maintaining authentication, confidentiality and integrity of clinical operations:

- *Device transparency:* The security local modules operate as a transparent inline component—an invisible intermediary that maintains the original network settings of the medical devices. By avoiding modifications to IP addresses or device configurations, this bump-in-the-wire deployment aligns with best practices in commercial-grade NGIPS platforms [34], preserving device sercurity and ensuring uninterrupted clinical functionality.
- *Scalability and cost*: Each embedded security module operates autonomously, enabling distributed deployment across clinical zones. This modular and low-cost design supports progressive, large-scale rollouts and localized policy enforcement without network-wide reconfiguration, adapting to evolving threat landscapes [18,23].
- *Network segmentation:* Each IoMT device is logically isolated through its own embedded firewall module, enabling fine-grained policy enforcement per device. This architecture minimizes lateral threat propagation and enhances containment in case of a breach.
- *Mandatory human supervision:* In critical healthcare environments, all automatically generated firewall rules (e.g., those produced by AI-based analysis) must be reviewed and approved by authorized personnel prior to deployment, ensuring accountability and preventing unintended service disruption. This human-in-the-loop approach prevents disruptions to critical medical services and ensures accountability.
- *Secure and authenticated communication:* All interactions between embedded security devices and the central manager use encrypted channels with mutual authentication. This ensures both the authenticity of the communicating parties and the confidentiality and integrity of transmitted alerts and rule updates.
- *Alignment with regulations and best practices*: The architecture aligns with healthcare-specific cybersecurity standards, including HIPAA and HITRUST CSF [11,13], through features such as device-level micro-segmentation, granular access control, and secure, auditable decision-making [12,19,23].

### 3.2. Implementation

This section presents the practical implementation of the proposed security framework, with emphasis on the construction of its core components, including Raspberry Pi–based embedded security devices and centralized management services. The description encompasses the software stack, containerized services, authentication, confidentiality and integrity mechanisms, as well as the orchestration of alert processing and rule enforcement. Deployment-specific considerations and system assessment will be discussed separately in Section 4.

Based on the functional architecture described in the previous section, the system’s operation centers on the detection and mitigation of network threats as follows (see Figure 5): *(i)* embedded devices continuously monitor traffic and generate, if so, security alerts; *(ii)* the alerts are transmitted to the central manager over HTTPS channels, protected with device-specific cryptographic keys retrieved from Vault; *(iii)* upon reception, alerts are cryptographically verified for authenticity, parsed into structured data, and processed by a LLM to automatically generate proposed firewall rules; finally, *(iv)* the rules are stored in a database and deployed to the devices after human validation, thereby ensuring safety and traceability in critical medical environments.

As a summary, the following tools are utilized to implement the aforementioned functionality. The centralized manager (CM) and its supporting services are deployed as Docker containers (Docker 28.2.2), providing authentication through OpenLDAP (OpenLDAP v1.5.0), secure key management with HashiCorp Vault (HashiCorp Vault v1.17.0), and persistence via PostgreSQL (PostgreSQL v17). A Flask-based backend (Flask v2.3.2) exposes the API and orchestrates the workflow from alert reception to rule enforcement, while a React frontend (React v18.2.0) enables operators to monitor devices, review alerts, and supervise rule deployment. The embedded devices, built on Raspberry Pi, integrate Suricata (Suricata 6.0.10) for intrusion detection and nftables (nftables v1.0.6 (Lester Gooch #5)) for firewall enforcement, complemented by custom scripts that manage log monitoring, cryptographic protection of alerts, and secure communication with the centralized manager.

**Figure 6:**
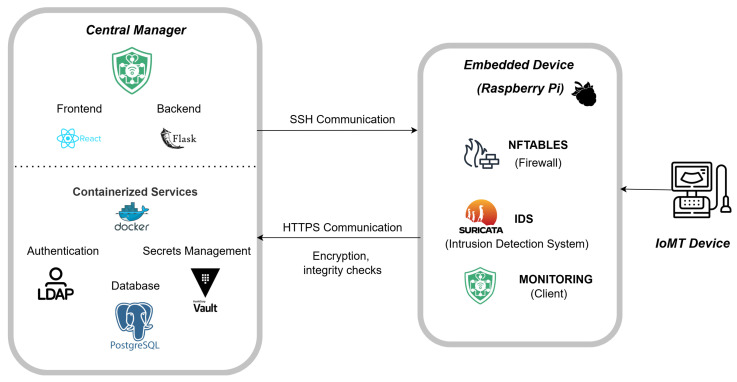
Tools, services, and components of *MEDIotWAL*.

Figure 6 highlights the main components, their interactions, and the secure communication flows between the centralized manager and the distributed devices in *MEDIotWALL*. All of them are described in the following.

#### 3.2.1. Centralized Manager

The *Centralized Manager* (CM) acts as the core control hub of the system, coordinating interactions with distributed IoMT devices while enforcing security policies and ensuring reliable operation. It offers a unified interface for device administration, alert management, authentication, and secure key handling.

The CM integrates four core services that collectively sustain secure operation and reliable interaction with distributed IoMT devices:

- *Web service:* This is the main interface between the CM and the IoMT devices. It exposes a REST API for communication, validates cryptographically protected alerts, and coordinates device registration and firewall rule management. The service is implemented with Flask, its frontend and backend-related functionalities being described below.
- *Database:* This component provides the persistent data layer of the system. A PostgreSQL instance stores critical information such as device metadata, alert histories, and firewall rules with their associated lifecycle states.
- *Authentication:* This service delivers centralized identity and access management. Built on OpenLDAP, it maintains user credentials and enforces group-based access control, enabling scalable administration of authorized users. By decoupling policy management from backend configuration, it supports flexibility and seamless adaptation as the system grows.
- *Key management:* This service ensures the protection of sensitive cryptographic material, including private keys and credentials. HashiCorp Vault is employed to securely store and manage these assets while enforcing fine-grained access policies. Its *AppRole* mechanism issues short-lived credentials that minimize long-term exposure and strengthen incident response capabilities by allowing rapid revocation and rotation when necessary.

The following sections detail its main components, emphasizing their roles in secure communication, access control, and system orchestration.

##### CM Frontend

The frontend constitutes the user-facing layer of the CM, implemented as a web application accessible via a secure HTTPS domain. It offers administrators a convenient and intuitive entry point to the system, eliminating the need for direct interaction with the underlying API or containerized services.

The interface is primarily designed to consolidate security information, streamline device and policy administration, and enhance usability in daily operations. In addition to its functional capabilities, the graphical design of the CM frontend significantly contributes to operational efficiency. Through the use of clear dashboards, visual indicators, and intuitive layouts, the interface reduces cognitive load for administrators, enabling rapid anomaly detection, response prioritization, and effective oversight of critical security events. This focus on usability and visual clarity ensures that even complex workflows—such as alert review or firewall rule validation—remain accessible and manageable in real-world healthcare environments.

**Figure 7:**
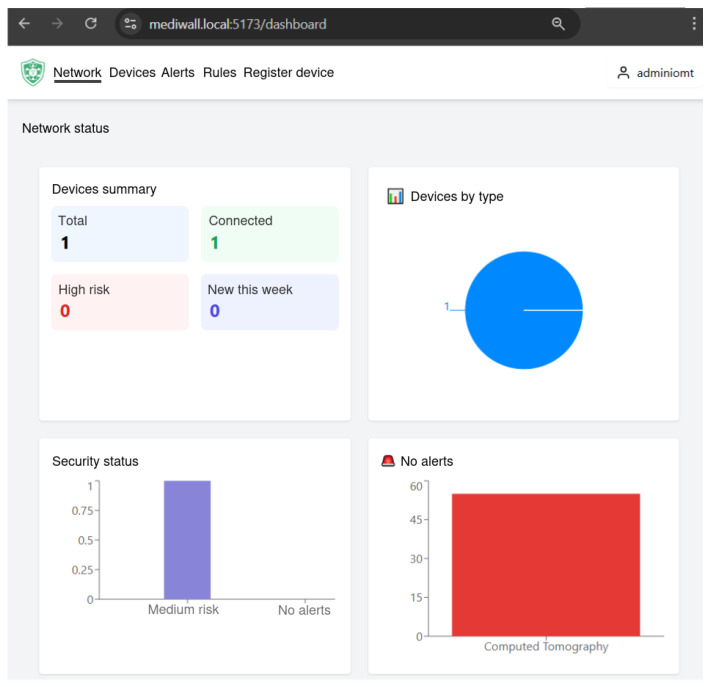
Main CM dashboard summarizing devices, alerts, and security status.

To illustrate its practical operation, the frontend is presented through several representative screenshots. Figure 7 displays the main dashboard, which consolidates the status of connected devices, security alerts, and system’s security state into a single view. This visualization enhances situational awareness by enabling administrators to assess the overall security posture at a glance.

**Figure 8:**
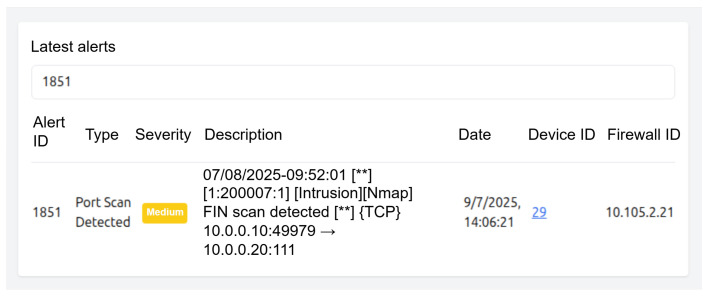
Alert interface showing an attack event detected in experimentation.

Figure 8 shows the alert management interface, where validated alerts from distributed devices are displayed chronologically, with filtering and search capabilities to locate specific events. Beyond raw data, the interface provides statistical summaries and graphical representations that support rapid assessment of incident trends and attack patterns. This dual perspective—both detailed and aggregated—enables thorough incident analysis as well as high-level strategic monitoring.

**Figure 9:**
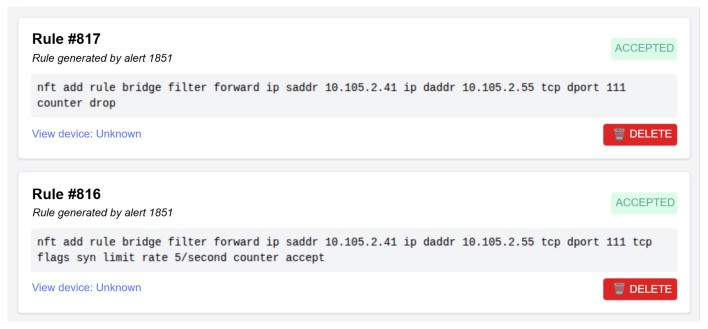
Firewall rules generated and applied in response to a given detected attack.

Figure 9 illustrates the rule management interface, where firewall rules automatically generated by the backend are displayed in prioritized order, with pending items highlighted for administrator review. This workflow integrates automation with human oversight, ensuring that final enforcement decisions remain under administrator control.

**Figure 10:**
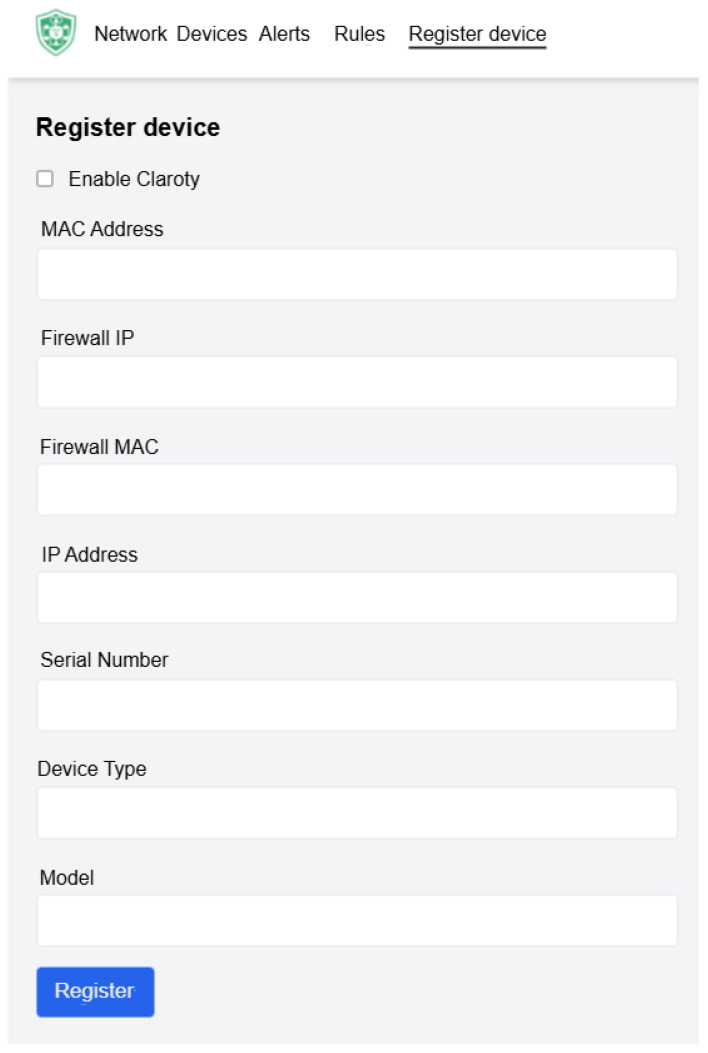
Device registration panel supporting manual input and Claroty-assisted onboarding.

Figure 10 shows the device registration panel, which centralizes the onboarding of new assets. Operators may either manually enter device information or enable Claroty-assisted registration, where metadata is automatically retrieved from the inventory. This approach minimizes configuration errors and accelerates device integration.

**Figure 11:**
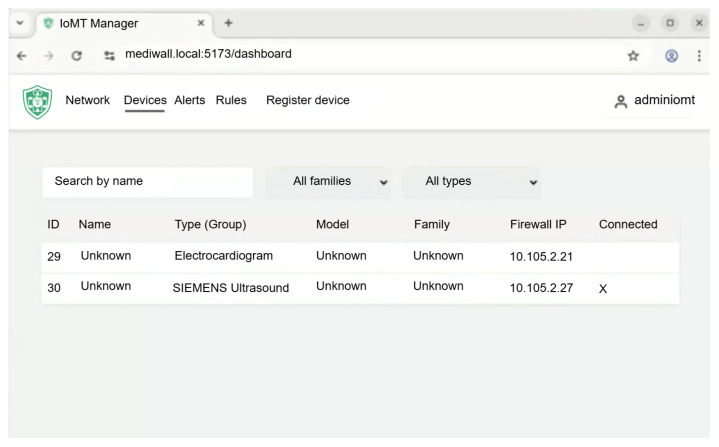
Device management panel with the registered IoMT nodes and their status.

Finally, Figure 11 shows the device management panel, providing detailed information on each registered IoMT node, including identifiers, network parameters, and associated policies. This interface supports both asset inventory and operational review during the analysis of past security events.

Beyond its functional role, the CM frontend incorporates security measures consistent with those enforced at the backend. Access to the web panel is restricted to authorized domains and delivered exclusively over HTTPS, ensuring the confidentiality and integrity of the operator sessions. Additionally, session cookies are configured with *Secure* and *HttpOnly* flags, while cross-origin requests are allowed only from predefined, trusted sources via strict CORS policies. These measures extend the system’s defense-in-depth strategy to the user interface, ensuring that administrators interact with the platform in a secure and controlled environment.

Equally important, the frontend is designed with usability as a cornerstone for secure and efficient operation. Through intuitive navigation, consistent representation of device and rule states (e.g., active/inactive, pending/approved/rejected), and immediate feedback for critical actions, the interface reduces operator cognitive load and minimizes the risk of configuration errors. This design philosophy reinforces the system’s human-in-the-loop approach, ensuring that automation is balanced with clear oversight and informed decision-making by security administrators.

Finally, the interface incorporates advanced visualization features that extend beyond raw data presentation. Dynamic dashboards and graphical summaries offer aggregated views of system status, including trends in alert frequency, severity distributions, and rule lifecycle states. These visual analytics complement detailed event listings, enabling administrator/s to quickly identify anomalies, recognize attack patterns, and prioritize responses effectively. By combining granular inspection with high-level situational awareness, the CM frontend enhances both tactical responsiveness and strategic monitoring in medical IoMT environments.

##### CM Backend

The backend of the Central Manager forms the operational core of the system. It orchestrates the REST API, enforces authentication mechanisms, and processes alerts encrypted with device-specific keys from IoMT devices. Additionally, it automates firewall rule generation using AI language models, manages policy enforcement and rule lifecycles, and provides auditing capabilities to ensure accountability and secure operation.

To achieve these objectives, the backend’s functionality is organized into eight core processes that collectively ensure secure operation and system adaptability. Each process is described below, emphasizing its role in communication, device management, or policy enforcement within the IoMT security architecture:

1. REST API orchestration: The backend provides a set of REST endpoints (implemented in Flask) that serve as the primary communication interface between embedded devices, security operators, and internal services. These endpoints support core functions such as device registration, reception of security alerts, firewall rule management across workflow states (pending, approved, rejected), and administrator authentication. All requests undergo strict validation and authentication to prevent unauthorized access or propagation of malformed data.
  The modular design also supports extensibility, allowing the integration of additional device types, intrusion detection systems (e.g., Suricata, Snort), or logging frameworks (e.g., Syslog, Wazuh, ELK) without altering the core backend.
2. Session and access control: Administrative access is enforced through authenticated sessions over HTTPS. User credentials are validated against the centralized LDAP directory, ensuring that only directory-registered accounts can log in. Session cookies are also configured with security flags (*HttpOnly, Secure, expiration*). Access to sensitive endpoints—including firewall rule management, device registration, and alert handling—is restricted to authenticated administrator/s, ensuring that only authorized personnel can perform critical operations.
3. Device registration: The backend incorporates a structured registration workflow that guarantees the secure onboarding of new IoMT devices. The registration process supports both manual enrollment and automated discovery via the specialized industrial cybersecurity platform Claroty, for asset visibility and threat management, ensuring that each device is provisioned with unique credentials, network parameters, and security policies required for safe integration. The process can be grouped into four main phases:
  - *Parameter acquisition and enrichment:* Devices provide identifiers such as MAC address and optional metadata. When available, additional information is automatically retrieved from the Claroty API (e.g., type, model, IP addresses, VLAN), complementing the record with some relevant network parameters.
  - *Cryptographic protection:* Critical fields (e.g., IP addres, VLAN, MAC) are encrypted using Fernet before storage. Additionally, SHA-256 hashes of these identifiers are computed to enable efficient device lookup and authentication when processing incoming alerts.
  - *Secure persistence:* Enriched and protected records are stored in the system database, ensuring data consistency and traceability. During alert processing, the hashed identifiers from the alert (e.g., IP, MAC) are matched against the database to recover the corresponding device ID and retrieve its public key from Vault for cryptographic verification of the alert.
  - *Credential provisioning:* Each device is assigned a unique SSH key pair, with private keys securely stored in a centralized vault and isolated per device. This approach ensures confidentiality, prevents cross-device credential exposure, and supports auditability and secure lifecycle management.
  As a part of the registration process, each device is asigned unique Vault credentials, the generation and delivery of which is detailed in the “Secret management” process below.
4. Secret management: A central pillar of the backend architecture is its integration with HashiCorp Vault, which provides centralized and secure management of credentials for both devices and the manager. Vault is deployed as an independent container and enforces fine-grained policies and authentication mechanisms tailored to distributed IoMT environments. The integration covers four main aspects:
  - *Secure key provisioning:* During device registration, each device is assigned a unique SSH key pair. The private key, protected with a passphrase, is securely stored in Vault under a device-specific path, while the public key remains available for verification. This avoids hardcoded credentials and enables subsequent key rotation.
  - *Policy-based access control: *Vault automatically issues per-device policies granting exclusive access to their own secrets, strictly following the principle of least privilege and preventing lateral credential exposure.
  - *Authentication:* Devices authenticate through Vault’s AppRole mechanism, which exchanges identifiers for short-lived tokens limited to the assigned policy. This mechanism is first used during device registration to retrieve their unique credentials and is subsequently reused for secure interactions with the backend, ensuring continuous policy enforcement.
  - *Isolation and auditability:* All interactions between the backend and Vault occur over an internal Docker network with encryption in transit and at rest. This setup supports immediate revocation, periodic rotation of credentials, and detailed auditing of all secret-related operations, enhancing resilience and compliance to medical IoMT deployments.
5. Rule generation with LLMs: To accelerate firewall rule creation and maintain adaptability in heterogeneous IoMT environments, the system leverages Large Language Models (LLMs) via the OpenRouter API to synthesize candidate rules from structured alert data (For the experiments described in Section 4, we used the shisa-ai/shisa-v2-llama3.3-70b:free model.). Only alerts that have been successfully decrypted and cryptographically verified using device-specific keys, and matched against device records in Vault, are used as input—ensuring all automated actions are based on trustworthy events.
  The LLM framework automatically tailors rules to the characteristics of each detected event, providing immediate proposals while maintaining auditability and traceability. During development, multiple LLMs were evaluated to select the model producing the most syntactically correct and semantically coherent firewall rules.
  The rule generation procedure is as follows:
  - *Automated rule synthesis:* Verified alerts are converted into a standardized JSON format that captures source and destination addresses, affected services or ports, and event type or severity. This structured representation is incorporated into a dynamic prompt that guides the LLM in generating firewall rules in nftables format). The generated rules initially take the form of abstract templates with placeholders (e.g., src_ip, dest_port), which are subsequently populated with actual alert values.
  - *Human-in-the-loop validation:* Candidate rules are stored in a pending state and reviewed by the administrator/s. Only approved rules are deployed, preserving operational control, security, and compliance with regulatory requirements.
6. Policy enforcement: The backend implements a controlled lifecycle for firewall rules, encompassing three primary actions:
  - *Approval and deployment:* Once a rule has been reviewed and accepted by an administrator, it is securely deployed to the target embedded device. Deployment occurs through an encrypted SSH connection using a private key dynamically retrieved from a centralized secret vault. Prior to execution, the system verifies the existence of required nftables tables and chains (input, forward, and output), creating them if absent, and selects the appropriate network family (*ip*, *inet*, or *bridge*) to ensure compatibility with the device environment.
    Commands are executed on the remote firewall, and upon success, the rule’s status is updated to ‘accepted’, and the timestamp of deployment is recorded, providing full auditability.
  - *Rejection:* The administrator/s can explicitly reject rules considered inappropriate, redundant, or unsafe. Rejected rules remain in the database with a rejected status and timestamp, preserving historical traceability. No changes are made to the firewall during rejection, guaranteeing that only vetted rules impact the network. This mechanism ensures a strict human validation step while maintaining a comprehensive audit trail.
  - *Controlled removal:* The system allows manual deletion of firewall rules to retire obsolete policies, correct misapplied rules, or perform maintenance. For rules in the ‘accepted’ state, deletion triggers removal from both the database and the remote firewall using secure SSH execution. For ‘pending’ or ‘rejected’ rules, deletion affects only the database entry. All deletion actions are logged irreversibly, including timestamps.
7. Alert verification and processing: The backend enforces a secure, end-to-end workflow for handling alerts from IoMT devices. This workflow integrates secure verification and structured data processing:
  - *Key provisioning:* Each device receives a unique SSH key pair, with private keys confined to the device and public keys securely stored in HashiCorp Vault, avoiding shared credentials.
  - *Alert reception and cryptographic verification*: Alerts are transmitted from devices via HTTPS endpoints. Instead of using formal digital signatures (e.g., X.509 or COSE), each device encrypts and authenticates its alert payload by using its device-specific key pair securely stored in Vault. Only alerts that successfully pass cryptographic verification are accepted into the processing workflow, effectively preventing unauthorized injection or tampering by non-registered devices.
    Upon reception, the backend: *(i)* matches hashed device identifiers from the alert (e.g., IP and MAC) against the database to retrieve the corresponding device ID; *(ii)* obtains the device’s public key from Vault; and *(iii)* use this public key to decrypt and cryptographically verify the alert payload, ensuring it was generated by the corresponding device and has not been tampered with.
  - *Structured storage:* Verified alerts are parsed into a standardized JSON format capturing source/destination addresses, affected services or ports, and event severity. This structured representation ensures that alerts can be reviewed and traced during operational monitoring.
8. Administrator notification: To enhance situational awareness and ensure human oversight, the backend integrates an *automated notification* mechanism that informs the security administrator/s whenever a new firewall rule enters the review phase. Upon generation of a candidate rule by the LLM, the system immediately dispatches an email to the designated administrator, including the rule identifier and a concise description.
  The notification system is designed to support *flexible configuration* for different deployment environments, allowing administrator/s to customize sender and recipient addresses, mail servers, ports, and authentication credentials through environment-specific settings. This ensures timely delivery of alerts while maintaining operational security.
  Moreover, all notification events, including dispatch attempts and delivery status, are logged to provide full *auditability and traceability*. This guarantees that no automated rule changes go unnoticed and that all human interventions prior to rule deployment are properly recorded.

##### Overall CM Workflow

As a summary of all the above, the overall workflow of the CM is as follows:

1. Distributed Raspberry Pi-based firewalls generate security alerts protected with device-specific cryptographic keys.
2. The CM receives the alerts via the REST API, authenticates them, and performs cryptographic verification.
3. Verified alerts are normalized, stored, and forwarded to the LLM for candidate firewall rule generation.
4. The administrator/s is notified, who must validate proposed rules (approve, reject, or remove).
5. Approved rules are securely deployed to embedded devices via SSH.
6. All operations are logged for auditability and compliance.

**Figure 12:**
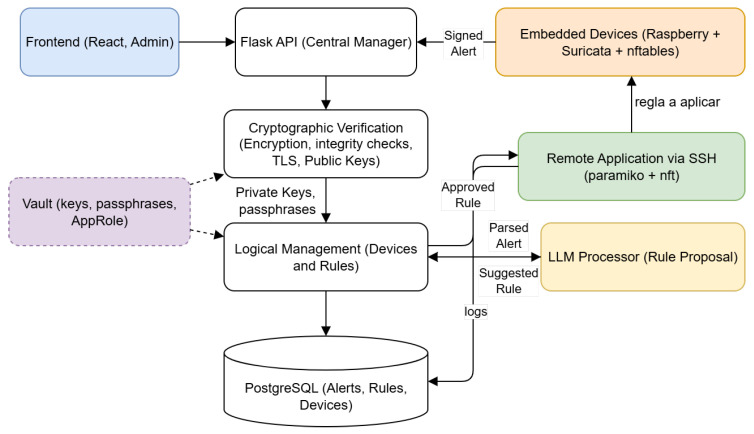
Overall CM workflow.

This sequence is illustrated in Figure 12, which highlights the generation of alerts, their cryptographic verification, and the process leading to candidate firewall rule creation and human validation, followed by secure deployment. It showcases the key interacting services—such as the Flask API, Vault, PostgreSQL, and the LLM engine—emphasizing the end-to-end integration that supports secure, auditable, and reliable rule enforcement across IoMT devices.

#### 3.2.2. Embedded Security Devices

Embedded security devices (ESDs) constitute the distributed enforcement layer of the *MEDIotWALL* architecture. Deployed inline between clinical equipment and the hospital network, each device functions as a transparent “bump-in-the-wire” security node. Its primary role is to provide localized monitoring and filtering without altering the IP configuration or certification status of the protected medical equipment.

Each ESD acts as both a sensor and an actuator within the overall security framework. On the one hand, it inspects traffic using an intrusion detection system (IDS) to identify suspicious behaviors or known attack signatures. On the other hand, it enforces filtering policies through a lightweight firewall that receives dynamic updates from the CM. Beyond traffic control, embedded devices also provide cryptographic assurance and system telemetry. All generated alerts are protected using device-specific cryptographic keys and transmitted securely to the CM, guaranteeing authenticity, confidentiality and integrity. Conversely, firewall rules and configuration updates are only accepted if transmitted over authenticated, encrypted channels, preventing tampering or unauthorized reconfiguration.

As previouly mentioned, ESDs are currently implemented on a low-power Raspberry Pi 5 with ARM architecture, running Raspberry Pi OS (Raspberry Pi OS (Debian GNU/Linux 12 “Bookworm”)), the lightweight official Linux-based distribution for this platform. This hardware has proven sufficient to support the aforementioned functionality, delivering stable performance in typical healthcare environments.

##### Firewall Functionality

The firewall ESDs component relies on nftables as the primary packet filtering framework. The selection of nftables is guided by its suitability with limited computational resources, offering a simplified configuration syntax, efficient rule management, and reduced overhead, making it particularly appropriate for resource-constrained platforms such as Raspberry Pi.

Unlike static configurations, rules are dynamically provisioned from the CM, ensuring that local filtering policies remain continuously aligned with the latest threat intelligence and contextual requirements of the monitored medical device.

Traffic filtering is enforced through fine-grained conditions, including source and destination addresses, transport protocols, and specific service ports. In addition, advanced features such as rate limiting, connection tracking, and counters are employed to mitigate common network attacks (e.g., flooding or brute-force attempts) without disrupting legitimate traffic. This allows administrator/s to enforce context-aware restrictions that adapt to both normal hospital traffic patterns and emerging anomalies.

From an operational perspective, firewall rules are transmitted securely over authenticated channels and applied via automated scripts executed through SSH with device-specific credentials. Before deployment, the system verifies the existence of required nftables tables and chains, creating them if absent, to ensure consistency across heterogeneous devices. This remote orchestration guarantees that local firewalls remain synchronized with centralized policies, while the transparent inline placement of the embedded device preserves the original network configuration and avoids interference with medical device certification.

##### Intrusion Detection on ESDs

For network-based intrusion detection, the ESDs integrate Suricata, an open-source IDS widely adopted in critical infrastructures. Even though *MEDIotWALL* does not depend on a particular option, Suricata is chosen instead of other solutions like Snort because it offers greater stability and a more straightforward deployment on ARM-based devices. While Snort is a widely used IDS, some reports highlight deployment challenges and additional complexities when running it on Raspberry Pi [39]. In contrast, Suricata has been successfully employed in recent studies for monitoring IoT networks on Raspberry Pi, demonstrating its suitability for embedded environments [40].

The baseline rule set covers common attacks such as denial-of-service attempts, port scans, and protocol-specific exploits that are particularly relevant in medical network environments. In addition, tailored rules are developed to capture traffic patterns and threats specific to IoMT devices deployed in hospitals, which increases detection accuracy beyond generic signatures. Configuration parameters such as alert thresholds are further adapted to the expected communication behavior of hospital networks, reducing the occurrence of false positives while preserving sensitivity to genuine anomalies.

Importantly, detected alerts are not handled as passive notifications: they are cryptographically protected and transmitted to the CM, where they can dynamically trigger the enforcement of firewall rules on the ESDs. This design ensures that the IDS functions not as an isolated monitoring component but as an active element within the detection-and-response loop, enabling a responsive and adaptive defense mechanism.

##### Monitoring and Communication

In our implementation, each ESD continuously monitors local IDS-generated logs to detect suspicious activity or anomalous events in real time. Whenever an event matches the configured IDS rules, the device encrypts the alert using keys securely stored in the centralized secret management system, ensuring both authenticity, confidentiality and integrity of the transmitted security information. Encrypted alerts are then sent over secure HTTPS channels to the centralized manager, where the backend decrypts and cryptographically verifies the payload, stores the alerts persistently, and consolidates them into near real-time dashboards.

In addition to alert transmission, each ESD periodically reports its operational status—including connection and disconnection events—to the CM. This health-monitoring mechanism enables continuous supervision of IoMT devices, complementing real-time threat detection with robust infrastructure observability.

## 4. Deployment and Evaluation

This section describes the deployment of the proposed security solution in a realistic hospital environment under controlled conditions, as well as the methodology used to evaluate its detection and mitigation capabilities.

### 4.1. Experimental Scenario

**Figure 13:**
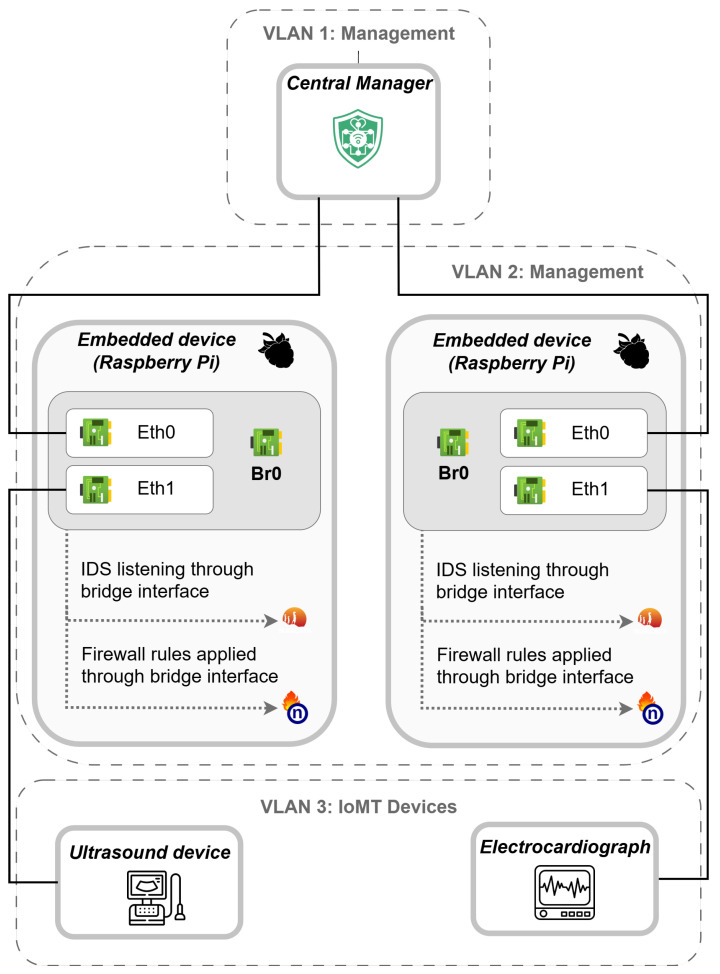
Experimental scenario to assess *MEDIotWALL*.

The experimental scenario is configured with the following key features (see Figure 13):

- The hospital network was conceptually segmented to separate management traffic from patient-care devices.
- A dedicated management VLAN interconnects the CM and the ESDs, while IoMT devices operate on isolated VLANs to limit lateral exposure.
- Each ESD is configured in bridge mode, linking the interfaces between medical equipment and the hospital network to preserve clinical workflow continuity.
- The bridge interface is assigned a static IP for communication with the CM, while the underlying physical interfaces operate in promiscuous mode to enable full traffic inspection.
- The CM was deployed as a virtual machine within the management VLAN.
- ESDs refer to Raspberry Pi 5 devices and FriendlyElec NanoPi R5C boards, each running the following:
  − Suricata IDS with an IoMT-specific rule set. To reduce false positives from routine broadcast and management traffic, alert thresholds in the IDS were tuned based on normal operational patterns. Specific parameter values are omitted to maintain network confidentiality.
  − The nftables firewall.
  − A custom monitoring client.
- Internet access for the ESDs was provided through the institutional proxy, supporting system updates and connectivity to the LLM API for proof-of-concept rule generation.
- As mentioned in the previous section, secure communications between the central manager and embedded devices were ensured using SSH with public/private key authentication.
- As IoMT devices, two representative medical devices are used: a *BeneHeart R12* electrocardiograph (ECG) and a *Siemens ultrasound* machine (UM).

### 4.2. Operation and Evaluation

In order to ensure a systematic assessment procedure, the experimental workflow was structured as follows:

1. Establishment of the physical connectivity among all elements: CM, ESDs and ECG/UM IoMT devices.
2. Configuration of the network aspects, including bridge mode operation for the ESDs.
3. Registration of the ESDs in the CM.
4. Deployment of initial configuration for the ESDs: Suricata, nftables, and the monitoring client adapted for bridge mode operation.
5. Running Suricata and the monitoring client on all ESDs.
6. Generation of controlled network traffic and simulated attacks from a dedicated host, targeting the IoMTs through the ESDs in a controlled manner.
7. Verification that alerts generated by the ESDs are received by the CM.
8. Reviewing and approval of automatically suggested firewall rules through the manager interface.
9. Application of the approved rules to the ESDs from the CM.
10. Confirmation that the firewall rules have been correctly enforced on the ESDs.
11. Repetition of the controlled attacks to validate mitigation effectiveness, monitoring packet drops and responses.
12. In a controlled environment with a simulated device, the system’s performance was further evaluated by measuring CPU and RAM usage at the ESDs, as well as the estimated end-to-end latency from detection to rule enforcement during systematically reproduced attacks.

To evaluate the proposed framework under realistic IoMT threat conditions, a set of controlled attacks were executed against the UM and ECG IoMTs considered. The attacks were selected to cover both volumetric denial-of-service attempts and reconnaissance scans, two of the most common categories of threats in healthcare networks. (The TCP SYN flood attack exemplifies a volumetric denial-of-service attempt, which is one of the most common threats to the availability of medical devices in hospital networks. In contrast, the Nmap-based reconnaissance scans, including the NULL scan, the FIN scan, and the OS fingerprinting attempts, represent stealthier adversarial actions aimed at service enumeration and operating system inference, which are typically used as a precursor to more targeted exploits.)

- Electrocardiograph (ECG): Two attacks against ECG were implemented as follows:
  − A TCP SYN flood using hping3 against port 481 of the device, representing a volumetric denial-of-service scenario.
  − Multiple reconnaissance scans with nmap, including Null scans, FIN scans, and OS fingerprinting, simulating adversarial attempts to enumerate services and infer OS characteristics.
- Ultrasound machine (UM): For the UM, the experimental focus was on targeted reconnaissance through OS fingerprinting with nmap. This scan aimed to identify exposed services and gather system information in order to simulate the early stages of a targeted attack campaign.

### 4.3. Results and Discussion

The experimentation confirmed that the proposed system is able to both detect and mitigate the executed attacks in real time. The following summarizes the main findings for each IoMT.

Table 1 and Table 2 provide detailed evidence of the system’s ability to detect and mitigate representative network threats in IoMT environments, where all IP addresses have been anonymized for confidentiality. In all cases, the system reports alerts for the executed attacks and proposes filtering rules to block them in real time.

The previous efficacy-related evaluation was complemented with operational metrics regarding CPU/RAM usage and end-to-end latency between alert detection and rule enforcement. For that, we instrumented a lightweight monitor on the ESDs, with the following results. With respect to CPU and RAM usage, Suricata maintained stable levels (0% CPU and 70 MB RAM), whereas Python3 (v3.11) exhibited spikes ranging from 22% to 174% CPU and 73–105 MB RAM, primarily due to the execution of monitoring scripts and the limited optimization of their connections. Although total ESD CPU usage peaked 93%–100% during alert processing, these spikes were short-lived and do not indicate sustained overload. In terms of time consumption, the average reaction time from detection to rule application ranged from approximately 1.5 to 38 s, depending on the number of alerts sequentially processed.

Most existing Raspberry Pi-based approaches [35,36,37,38] focus on conceptual architectural security, network segmentation, or basic detection, without reporting measurable performance or active mitigation. This way, although the literature lacks broadly comparable quantitative results from alternative solutions, the findings here allow us to conclude that *MEDIotWALL* can effectively mitigate network- and transport-layer attacks by exploiting abnormal TCP header patterns and connection behaviors, while sustaining acceptable overall performance at low cost.

Despite the abovementioned promising results, the following limitations must be acknowledged:

- The mitigation mechanisms rely primarily on source-IP based filtering and simple rate-limiting. While this approach is effective for single-source attacks, distributed attacks or source address spoofing would require more advanced correlation mechanisms and group-based rule enforcement.
- EDSs now constitute potential failure points, since a device crash would imply the loss of connectivity for the associated medical device. Such an undesirable loss of connectivity in medical devices could be automatically detected by implementing a simple neighborhood procedure, in which the CM and each EDS periodically exchange accessibility messages.
- The current system focuses exclusively on network- and transport-layer attacks; application-layer exploits (e.g., SQL injection, XSS on web-based medical interfaces), encrypted traffic, and insider threats remain outside its detection scope. Furthermore, the current evaluation does not include advanced evasion techniques, which will be considered in future work.
- The implementation of *MEDIotWALL* employed an external LLM API for rule generation; however, in real IoMT deployments, a locally trained should be preferable. Due to privacy concerns and the risk of potential data leaks associated with proprietary LLMs typically provided via external APIs, locally deployed open-source LLMs on an infrastructure controlled by healthcare institutions are recommended, as discussed in [41].

## 5. Conclusions

This work introduces *MEDIotWALL*, a low-cost two-level distributed firewall-based security solution to protect healthcare environments. Compared to other existing IoMT security solutions, the proposed approach emphasizes practical deployment, device-level protection, and minimal infrastructure overhead.

At present, commercial platforms generally require centralized management and dedicated network integration, whereas traditional enterprise firewalls provide only perimeter-level protection. By contrast, the proposed bridge-mode deployment using embedded devices offers transparent, per-device security without modifying existing medical equipment or hospital network workflows, making it particularly suitable for legacy IoMT environments. The integration of open-source IDS and firewall components ensures cost-effectiveness, transparency, and reproducibility. Additionally, the system supports administrator-supervised, automated rule generation via LLMs, enabling dynamic and rapid mitigation of network-level threats while maintaining auditability and human oversight. While other available IoMT security-related solutions on embedded platforms focus on detection efficacy, we also report CPU/RAM usage and alert-to-rule latency, providing operational insights for deployment on low-cost, resource-constrained devices.

As future research directions, Section 4.3 points out some relevant aspects, like extending the system to more diverse and complex IoMT-related deployments, application-layer detection, integrating additional log sources for correlation, assessing resilience against distributed attacks. Moreover, because protocols such as DICOM/HL7 (Digital Imaging and Communications in Medicine/Health Level 7) are complex and require specialized contextual knowledge, general Suricata and nftables rules may not be sufficient to detect all vulnerabilities. For more comprehensive security, DICOM/HL7-aware rules or fuzzing techniques should be considered in future research and deployments.

## Figures and Tables

**Table 1:** Alert and rule details for the attacks against the ECG.

**Attack: TCP SYN flood**	hping3 -S -p 481 –flood 10.0.0.20
**Alert**	07/08/2025-09:15:44.440308 [**] [1:200002:2] [DDoS][IOMT] Unusually high traffic [**] TCP 10.0.0.10:3837 → 10.0.0.20:481
**Rules**	ip saddr 10.0.0.10 ip daddr 10.0.0.20 tcp dport 481 tcp flags syn limit rate 5/second counter packets 22 bytes 1012 accept ip saddr 10.0.0.10 ip daddr 10.0.0.20 tcp dport 481 tcp flags syn counter packets 109,772 bytes 5,049,512 drop
**Packets blocked/accepted**	109,772 blocked/22 accepted
**Attack: Nmap NULL scan**	nmap -sN 10.0.0.20 -p 21,22,80,443,1720
**Alert**	07/08/2025-09:24:32.288151 [**] [1:200006:1] [Intrusion][Nmap] NULL scan detected [**] [Classification: (null)] [Priority: 3] TCP 10.0.0.10:39515 → 10.0.0.20:21-443,1720
**Rules**	ip saddr 10.0.0.10 ip daddr 10.0.0.20 tcp dport [21/22,80,443,1720] tcp flags syn limit rate 5/second counter acceptip saddr 10.0.0.10 ip daddr 10.0.0.20 tcp dport [21/22,80,443,1720] counter drop (Rules separated per port)
**Packets blocked/accepted**	2 blocked per port (total 10)/0 accepted
**Attack: Nmap FIN scan**	nmap -sF 10.0.0.20 -p 111
**Alert**	07/08/2025-09:52:01 [**] [1:200007:1] [Intrusion][Nmap] FIN scan detected [**] TCP 10.0.0.10:49979 → 10.0.0.20:111
**Rules**	ip saddr 10.0.0.10 ip daddr 10.0.0.20 tcp dport 111 tcp flags syn limit rate 5/second counter accept ip saddr 10.0.0.10 ip daddr 10.0.0.20 tcp dport 111 counter drop
**Packets blocked/accepted**	2 blocked/0 accepted
**Attack: Nmap OS fingerprinting and high traffic (DDoS)**	nmap -O –reason 10.0.0.20
**Alert**	07/15/2025-16:42:03 [**] [1:300005:1] [Intrusion][Nmap] Suspicious OS fingerprinting (SYN without payload) [**] TCP 10.0.0.10:45971 → 10.0.0.20:1720 07/15/2025-16:42:04 [**] [1:200002:2] [DDoS][IoMT] Unusually high traffic [**] TCP 10.0.0.10:45973 → 10.0.0.20:3920
**Rules**	ip saddr 10.0.0.10 ip daddr 10.0.0.20 tcp dport 1720 tcp flags syn limit rate 5/second counter accept ip saddr 10.0.0.10 ip daddr 10.0.0.20 tcp dport 1720 counter drop ip saddr 10.0.0.10 ip daddr 10.0.0.20 tcp dport 3920 tcp flags syn limit rate 5/second counter accept ip saddr 10.0.0.10 ip daddr 10.0.0.20 tcp dport 3920 counter drop ip saddr 10.0.0.10 ip protocol tcp counter drop
**Packets blocked/accepted**	2012 blocked/0 accepted

**Table 2:** Alert and rule details for the attack targeting the UM.

**Attack: Nmap OS fingerprinting**	nmap -O –reason 10.0.1.20
**Alert**	07/17/2025-11:09:XX [**] [1:300005:1] [Intrusion][Nmap] Suspicious OS fingerprinting (SYN without payload) [**] TCP 10.0.1.10:XXXXX → 10.0.1.20:1720/5060
**Rules**	ip saddr 10.0.1.10 ip daddr 10.0.1.20 tcp dport 1720 tcp flags syn limit rate 5/second counter accept ip saddr 10.0.1.10 ip daddr 10.0.1.20 tcp dport 1720 counter drop ip saddr 10.0.1.10 ip daddr 10.0.1.20 tcp dport 5060 tcp flags syn limit rate 5/second counter accept ip saddr 10.0.1.10 ip daddr 10.0.1.20 tcp dport 5060 counter drop
**Packets blocked/accepted**	2030 blocked/0 accepted

## Data Availability

The datasets referenced in this article are not publicly available due to the sensitive nature of the specific healthcare environment involved. Requests for access to the datasets should be directed to the Andalusian Health Service (SAS).

## Abbreviations

The following abbreviations are used in this manuscript:

API	Application Programming Interface
CM	Central Manager
CSF	Common Security Framework
DDoS	Distributed Denial of Service
ECG	Electrocardiograph
ESD	Embedded Security Device
HDO	Healthcare Delivery Organizations
H-IoT	Healthcare Internet of Things
HIPAA	Health Insurance Portability and Accountability Act
HITRUST	Health Information Trust Alliance
IDC	International Data Corporation
IDPS	Intrusion Detection and Prevention System
IoMT	Internet of Medical Thing
IoT	Internet of Things
IP	Internet Protocol
LLM	Large Language Model
MRI	Magnetic Resonance Imaging
OS	Operating System
PHI	Protected Health Information
SSH	Secure SHell
TCP	Transmission Control Protocol
UM	Ultrasound Machine
